# Custard apple seed induced keratitis: a harmful traditional practice in South India

**DOI:** 10.3205/oc000074

**Published:** 2017-09-01

**Authors:** Pratheeba Devi Nivean, S. Malarkodi, M. Nishanth, M. Nivean

**Affiliations:** 1 M.N. Eye Hospital, Chennai, India

**Keywords:** custard apple seeds, keratitis, harmful traditional practice

## Abstract

Custard apple seeds have been used as a traditional remedy for scalp infestation of lice and dandruff since many years in our part of the world. Not many people, doctors even, are aware about the ill effects of this practice. Surprisingly, there are very few reports on poisoning due to these seeds and the management is not very well streamlined. Rohit Shetty et al. highlighted the ill effects of the seeds and the need to avoid steroids in these cases as they worsen the clinical scenario.

We present 2 patients who developed keratoconjunctivitis due to ocular exposure with custard apple seeds. However, we managed our cases slightly differently. We scraped off the loose epithelium and patched the eye for a day or two with topical antibiotics and lubricants. The patients responded very well. We highlight our series to describe an alternate way of treatment for cases with custard apple seed injury.

## Introduction

Custard apples [Annona squamosa] are cultivated throughout India and are commonly known as “sitaphal” or “sugar apples” [1]. Their seeds (in powdered form) are used as a remedy for lice and dandruff in some parts of India. Their accidental contact with the eyes is not uncommon in South India. We present our experience with custard apple seed induced keratitoconjunctivitis following accidental ocular exposure.

## Case descriptions

### Case 1

**Figure 1:**
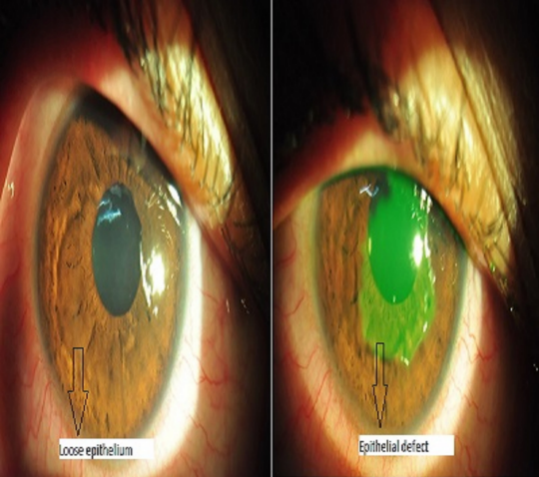
Slit lamp photo of the right eye showing large corneal epithelial defect with surrounding loose epithelium

**Figure 2:**
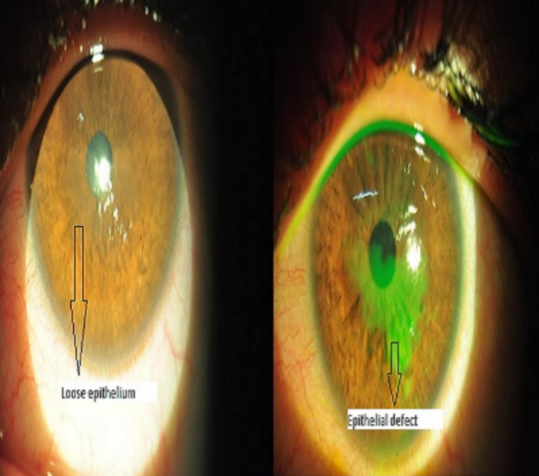
Slit lamp photo of the left eye showing corneal punctuate epithelial erosions, abrasions, and loose epithelium

A 21-year-old female presented to our hospital with complaints of inability to open both eyes, lid swelling, defective vision, ocular pain, watering, and redness since 3 days. On further questioning, she gave history of having applied custard apple seeds ground to a paste to her hair as a remedy for dandruff. She had been on treatment with an ophthalmologist who had prescribed topical antibiotics and lubricants. As there was no improvement with the above treatment, she came to our hospital for review 3 days later. On examination, her eyes were opened with difficulty and her visual acuity was 1/60 in both eyes. Slit lamp examination of the right eye after applying topical anaesthetic eyedrops revealed conjunctival congestion and a large epithelial defect with surrounding loose epithelium on the cornea (Figure 1 (Fig. 1)). The left eye revealed conjunctival congestion and corneal punctuate epithelial erosions, abrasions and loose epithelium (Figure 2 (Fig. 2)).

**Figure 3:**
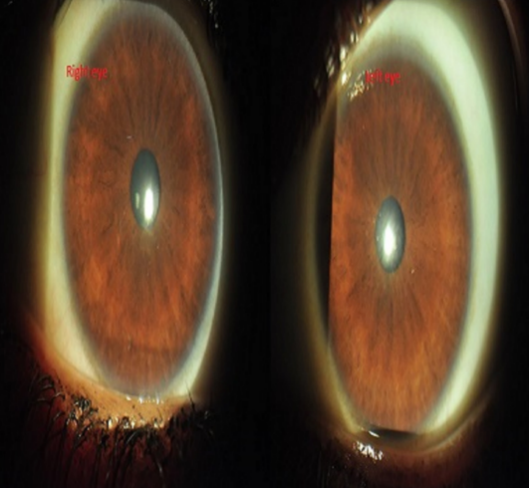
Slit lamp photo of both eyes after treatment

We scraped the loose epithelium in the right eye under topical anaesthesia. A rim of healthy epithelium was left behind for tissue regrowth and the eye was patched after applying topical antibiotic and lubricant eye gel. On the next day, vision in the right eye had improved to 6/18 and the epithelial defect had reduced. Patching was continued and on the 3^rd^ day, the defect had healed but a nebular haze was present. Lubricants were continued. The left eye was left un-patched prescribing her with a copious amount of lubricants alone on the first day. As there was no improvement seen the next day, scrapping of the epithelium was done in this eye too and patched for 2 days. On the 4^th^ day, she had a vision of 6/12 in both eyes and a fully healed epithelium with nebular corneal haze for which she was given mild steroids, fluromethalone and lubricating eye drops. She was examined again after a week when her visual acuity improved to 6/6, N6 in both eyes with a clear cornea (Figure 3 (Fig. 3)). Steroids were tapered and stopped and she was continued with lubricants alone. On follow-up after 6 months, her eyes were quiet with a vision of 6/6 in both eyes with no further sequelae. 

### Case 2

**Figure 4:**
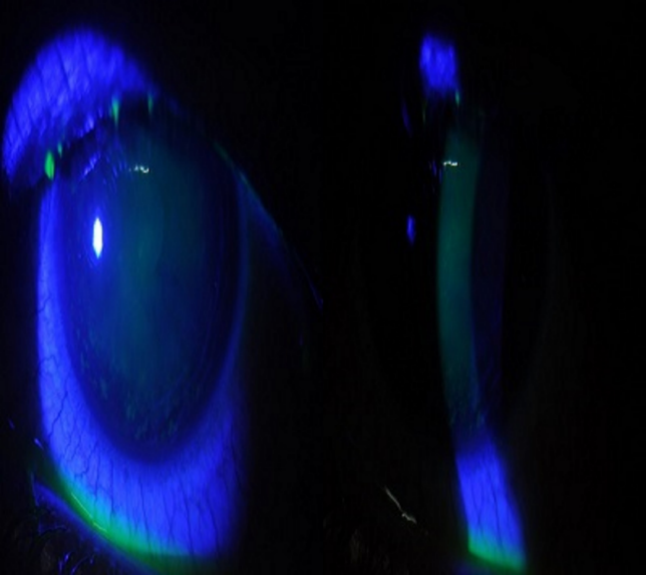
Slit lamp photo of the left eye showing loose epithelium with punctate erosions and abrasions

**Figure 5:**
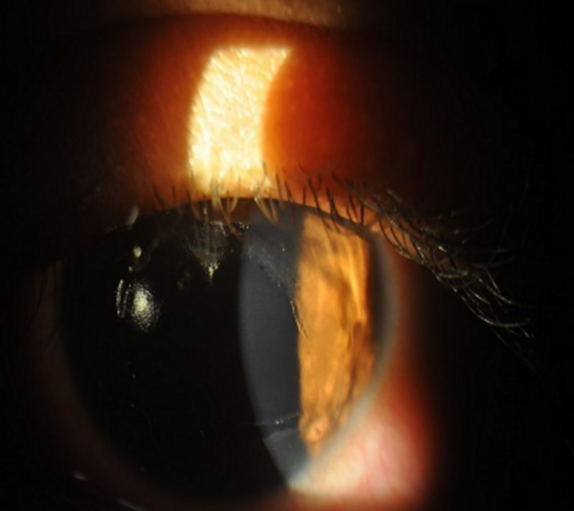
Slit lamp photo after scrapping of epithelium

**Figure 6:**
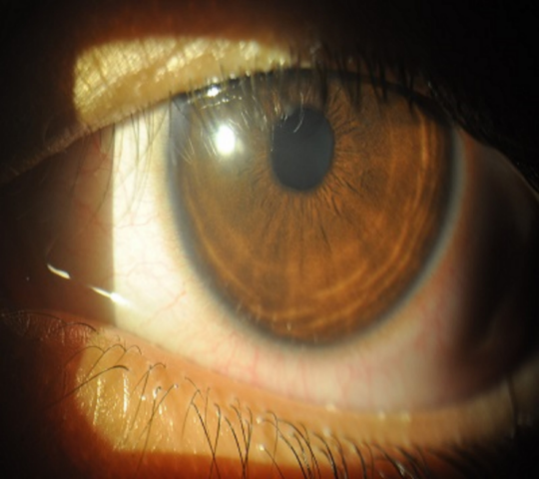
Slit lamp photo after treatment

A 13-year-old female came to us with complaints of irritation, redness, and watering in her left eye for 3 days. She gave history of washing her hair with powdered custard apple seeds as a remedy for lice after which she developed redness in her left eye. She had consulted someone else and was on antibiotics and lubricants. On examination, her vision was 6/6, N6 and 6/9, N6 in the right and left eye, respectively. Slit lamp evaluation of the left eye revealed loose epithelium with punctate erosions and abrasions (Figure 4 (Fig. 4)). Under topical anaesthesia the loose epithelium was scraped with sterile blade (Figure 5 (Fig. 5)) and the eye was patched after applying topical antibiotic and lubricating eye ointment. On review the next day, vision had improved to 6/6 and the epithelial defect had healed (Figure 6 (Fig. 6)). There was no haze. She was advised to continue lubricants for a week and asked for follow-up after 6 months.

## Discussion

There are very few reports explaining toxic effects following exposure of the eyes to custard apple seeds. Active compounds such as alkaloids, cyclohexapeptides, and acetogenins contained in the seeds cause abnormality in epithelial integrity [2]. To the best of our knowledge we found only one report by Nagaraja et al. [3]. 

Chemical injury due to the toxin can cause corneal erosions and abrasions. The pathogenic mechanism is related to poor adhesion of the corneal epithelium to the underlying stroma [4]. Excessive matrix metalloproteinase (MMP) activity may play a role in the pathogenesis [3]. Constant movement of the lids also contributes to increasing the abrasions. We found the epithelium to be loose in our cases so we scraped them with a sterile blade under slit lamp magnification and topical anaesthesia. We patched the eye with antibiotic drops and lubricating gel. Surprisingly, they all healed well within two days. None of our cases worsened or developed any kind of complications thereafter. Although there was no evidence to suggest an infectious aetiology, topical antibiotics were given to prevent any secondary infection that may occur. Lubricants helped in faster and better healing of the cornea. Patching the eye prevented continuous lid and eye movement.

Since the toxins affect the epithelium, removing the loose epithelium makes healing faster. As already documented by Rohit Shetty et al., steroids should be avoided in such cases in the acute phase before epithelial healing [3]. The endothelial study which we did for one of our cases was within normal limits suggesting there is no penetration of the toxin beyond the epithelium.

The common differential is viral keratoconjunctivitis. If the history is not properly elicited this condition can be maltreated. The treating physician should keep this condition in mind as the treatment is different. Steroids can worsen the situation. 

## Conclusion

We present our series to highlight the importance of this prevailing traditional practice. We present an alternate way of managing this problem. More public awareness of the harmful effects of the custard apple seeds should be created to avoid them. 

## Notes

### Competing interests

The authors declare that they have no competing interests.
